# Reversible atransferrinemia in a patient with chronic enteropathy: is transferrin mandatory for iron transport?

**DOI:** 10.11613/BM.2023.010801

**Published:** 2022-12-15

**Authors:** Alexandre Raynor, Carmen Stefanescu, Arnaud Bruneel, Hervé Puy, Katell Peoc’h, Hana Manceau

**Affiliations:** 1 Department of Biochemistry, Bichat University Hospital, APHP.Nord, Paris, France; 2 Department of Gastroenterology, Beaujon University Hospital, APHP. Nord, Clichy, France; 3 French Porphyria Center, Louis Mourier University Hospital, Colombes, France; 4 Department of Biochemistry, Beaujon University Hospital, APHP. Nord, Clichy, France; 5 Université Paris Cité, INSERM U1149, HIROS Heme Iron and Oxidative Stress, Inflammation Research Center, Paris, France

**Keywords:** transferrin, iron, inflammation, enteritis

## Abstract

Herein, we report the case of a 42-year-old woman, hospitalized in a French tertiary hospital for a relapse of a chronic enteropathy, who was found on admission to have no detectable serum transferrin. Surprisingly, she only exhibited mild anaemia. This atransferrinemia persisted for two months throughout her hospitalization, during which her haemoglobin concentration remained broadly stable. Based on her clinical history and evolution, we concluded to an acquired atransferrinemia secondary to chronic undernutrition, inflammation and liver failure. We discuss the investigations performed in this patient, and hypotheses regarding the relative stability of her haemoglobin concentration despite the absence of detectable transferrin.

## Introduction

In physiological conditions, iron, a vital metal constitutive of haemoglobin and haemoproteins, circulates in the bloodstream bound to transferrin (Tf). This complexation aims to protect the body from the adverse pro-oxidant effects of free iron, which can generate radical species through the Fenton reaction. Transferrin is a 79 kDa glycoprotein mainly synthesized in the liver, exhibiting two iron binding sites (*1*). The Tf-Fe^3+^ complex delivers iron to peripheral tissues through endocytosis *via* transferrin receptor 1 (TfR1) to meet their needs. This process is essential for erythroid cells, for which Tf is believed to be the sole provider of iron for haemoglobin synthesis *in vivo.* Since the seminal description in 1961, a handful of cases of congenital and acquired atransferrinemia have been reported in the literature (*2*). Here, we report the intriguing and rare description of a woman with a clinical picture of acquired atransferrinemia, with only mild clinical and haematological impacts. This observation raises significant questions on possible compensatory mechanisms for the iron supply of erythroblasts in human diseases. We discuss these possible mechanisms, including findings on potential alternatives to the Tf/TfR1 pathway.

## Materials and methods

### Subject

Informed consent was obtained from the patient whose case is reported herein. The subject was a 42-year-old woman hospitalized from April to September 2021 in a French tertiary university hospital (Beaujon University Hospital, Clichy, France) for the relapse of an unexplained chronic enteropathy characterized by stenosis and perforations. Clinical signs included unrelenting, non-radiating abdominal pain, nausea, vomiting, and diarrhea. The patient was malnourished, with a body mass index of 18.3 kg/m^2^. The history of her enteropathy included multiple acute episodes, leading to five surgical resections of the small intestine. Besides the enteropathy, she also exhibited Raynaud’s syndrome and miscarriage(s). Since adolescence, she experienced chronic iron-deficient anaemia without a clear etiology, repeatedly treated by red blood cell (RBC) transfusions. She underwent an anaphylactic shock in response to intravenous iron injection and was intolerant to oral iron supplementation.

### Methods

All clinical and biological data were obtained within the patient’s clinical course. Serum Tf was measured by immunoturbidimetry on an Abbott Architect (Abbott Laboratories, Chicago, USA) and nephelometry on a Siemens Vista Dimension (Siemens Healthineers, Erlangen, Germany). The limit of quantification was the same for both methods (0.09 g/L). Analysis of Tf glycoforms was performed by capillary electrophoresis on a Sebia Capillarys (Sebia, Monaco), following the manufacturer’s recommendations. Other routine biochemical parameters related to iron metabolism (iron, ferritin), hepatic function (aspartate aminotransferase (AST), alanine aminotransferase (ALT)), C-reactive protein (CRP), vitamins B9 and B12 were quantified on the Abbott Architect. Analyses of albumin and prealbumin were performed by nephelometry on an Atellica NEPH 630 (Siemens Healthineers, Erlangen, Germany). Soluble transferrin receptor 1 (sTfR1) was quantified on a Siemens Vista Dimension. Serum hepcidin was quantified with a previously published method of liquid chromatography coupled to tandem mass spectrometry (*3*). Analysis of faecal calprotectin was performed with a chemiluminescent immunoassay on a Diasorin LIAISON XS (Diasorin, Saluggia, Italy). Blood counts were performed on a Sysmex XE-2100 (Sysmex Corportion, Kobe, Japan) and coagulation factors were measured on a STA R Max 3 (Stago, Asnières-sur-Seine, France). At admission, the patient underwent 18-fluorodeoxyglucose positron emission tomography/computed tomography (18-FDG-PET/CT), colonoscopy and upper gastrointestinal endoscopy. Finally, as two of the patient’s sisters also experienced digestive symptoms, a genetic study was performed for the patient. This consisted in a targeted next-generation sequencing panel, as previously published (*4*).

## Results

### Biochemistry and haematology

Results of laboratory examinations on admission are reported in Table 1. Based on these results, the patient was found to suffer from malnutrition (decreased albumin, prealbumin), acute inflammation (increased CRP, faecal calprotectin, ferritin), liver failure (increased ALT, AST, decreased prothrombin time, factor V), and a mild, normocytic non-regenerative anaemia (decreased haemoglobin, normal reticulocytes). The exploration of iron metabolism evidenced an atransferrinemia, with a slightly lowered serum iron concentration. Plasma hepcidin was normal, and sTfR1 was moderately lowered.

**Table 1:** Laboratory examination results for the patient at admission

**Parameter**	**Result**	**Laboratory reference interval**
**Nutrition markers**		
Albumin (g/L)	13.1	40.6-49.6
Prealbumin (g/L)	0.032	0.195-0.338
**Inflammation markers**		
CRP (mg/L)	21	< 6
Faecal calprotectin (µg/g)	1111	< 50
**Iron status markers**		
Ferritin (µg/L)	2702	30-200
Serum iron (µmol/L)	5	10-30
Transferrin (g/L)	< 0.09	2.0-3.8
sTfR1 (mg/L)	0.47	0.76-1.76
Hepcidin (µg/L)	5	1-20
**Liver function tests**		
ALT (U/L)	228	< 34
AST (U/L)	70	< 31
**Blood count**		
Red blood cells (T/L)	3.7	3.93-5.09
Reticulocytes (G/L)	71	50-120
MCV (fL)	89	80-100
Haemoglobin (g/L)	105	115-149
**Coagulation**		
Prothrombin time (%)	27	70-120
Factor V (%)	37	70-120
CRP - C-reactive protein. sTfR1 - soluble transferrin receptor 1. ALT - alanine aminotransferase. AST - aspartate aminotransferase. MCV - mean corpuscular volume.		

**Figure 1:**
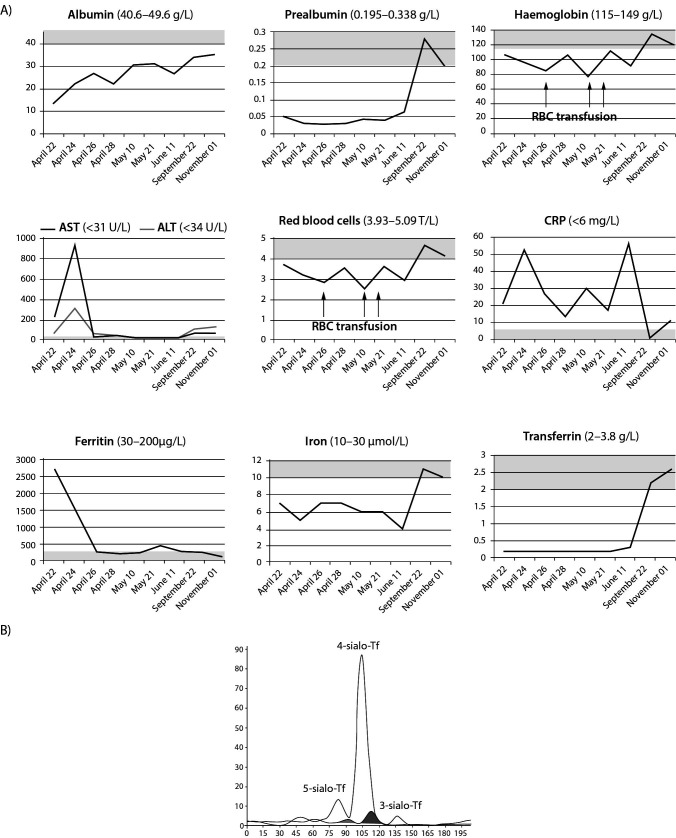
**A.** Evolution of serum markers during the course of the patient’s hospitalization. For each parameter, the laboratory reference interval is indicated between parentheses. **B.** Capillary zone electrophoresis of serum transferrin (Tf) glycoforms (5, 4 and 3-sialotransferrin). Overlay of Tf patterns after admission (black) and before discharge (white). RBC - red blood cells. Tf - transferrin. ALT - alanine aminotransferase. AST - aspartate aminotransferase. CRP - C-reactive protein.

Subsequent evolutions of these parameters are reported in Figure 1A. Enzymes ALT and AST increased significantly in the days following admission, at 936 and 314 U/L, respectively. The patient was transfused with RBC thrice (2, 1 and 2 packed RBC, respectively) when her haemoglobin concentration went < 80 g/L, given parenteral nutrition (including vitamins), albumin infusions to treat important edemas, and N-acetylcysteine. After that, hepatic transaminase activities normalized, but malnutrition, inflammation, and iron metabolism markers remained pathological. Vitamin B9 blood concentration was controlled as within the laboratory reference interval, while vitamin B12 concentration was increased, possibly due to excessive intravenous supplementation. Serum Tf remained undetectable for two months. However, the anaemia did not worsen, with the patient’s haemoglobin concentration fluctuating between 80 and 110 g/L, even after the end of transfusion support (Figure 1A).

Analysis of Tf by capillary electrophoresis revealed a very slightly altered distribution of glycoforms (5-sialo-Tf 20.4% [reference interval: 11-18%], 4-sialo-Tf 75.2% [78-86%], 3-sialo-Tf 3.9% [< 6%]) and revealed the presence of a trace concentration of Tf in the patient’s serum (Figure 1B).

Afterwards, the patient underwent a new resection of 60 cm of the small intestine, and her laboratory parameters subsequently normalized, leading to her discharge.

### Genetics

The targeted next-generation sequencing panel evidenced a heterozygous variant (c.1416-1417del) in the nuclear factor kappa b subunit 2 *(NFKB2)* gene. White blood cell immunophenotyping displayed a global lymphopenia (1.032 G/L (1.300-4.400)), albeit with normal subpopulations. Two heterozygous compound variants in the soluble carrier organic anion transporter family member 2A1 (*SLCO2A1)* gene were also identified: a premature stop codon (p.S550*), and a missense variant (c.1660G>A,p.G554R). *SLCO2A1* encodes a prostaglandin transporter notably associated with primary hypertrophic osteoarthropathy and hereditary chronic enteropathy (*5*).

### Imaging

Intestinal imaging did not evidence intestinal haemorrhage at admission. Computed tomography did not evidence peripheral iron overload.

## Discussion

The mainstay of this case report is the acquired atransferrinemia (confirmed by two different methods) in a patient presenting with a chronic enteropathy, but with only mild clinical and haematological repercussions. Congenital atransferrinemia was excluded in our patient due to the advanced age at presentation, the absence of iron overload in peripheral tissues as evidenced by computed tomography, and the normal serum hepcidin concentration. The most convincing argument was the subsequent normalization of serum Tf after the resolution of the acute episode. While anaemia linked to intestinal bleeding was reported as a clinical feature of *SLCO2A1* enteropathy, which was a discussed diagnosis for our patient, no such disruption of iron metabolism was previously described (*5*). Thus, we suspected in our patient a multi-etiological acquired atransferrinemia secondary to chronic undernutrition, inflammation, and liver failure. Interestingly, a trace amount of Tf was detectable by capillary electrophoresis; this is akin to patients with congenital atransferrinemia, which are known to possess residual concentrations of Tf, as a complete absence could be fatal (*6*). Furthermore, the electrophoretic profile did not reveal any structural abnormality or major glycosylation abnormality, which is important as we hypothesized that our chemistry analysers might have inadequately quantified an abnormal Tf.

Inflammation is usually associated with increased serum ferritin and hepcidin concentrations through the Interleukin-6/Janus kinase/Signal transducer and activator of transcription 3 (IL-6/Jak/STAT-3) pathway. However, despite acute inflammation, plasma hepcidin was normal in our patient, suggesting a low basal level related to iron deficiency. In the near-complete absence of Tf, we expected an increase in sTfR1 as a marker of iron deficiency in erythroblasts; however, sTfR1 was moderately lowered.

Surprisingly, our patient’s anaemia was only mild at presentation despite the atransferrinemia. Even more surprisingly, the anaemia was normocytic, whereas microcytosis would have been expected in the context of both nutritional and functional iron deficiency. This observation was possibly due to pre-existing vitamins B9/B12 deficiencies due to malnutrition. Persistant residual erythropoiesis was evidenced by a normal reticulocyte count, which was unexpected with an insufficient iron supply. Intestinal imaging did not evidence any apparent haemorrhage at admission.

Congenital atransferrinemia (OMIM #209300) is associated with genetic variants in the *TF* gene (3q22.1), usually associated with dramatic consequences related to iron overload in peripheral tissues and significant anaemia (*7*). The most recent case reported by Daboubbi *et al.* was a 6-month-old girl with severe hypochromic, microcytic, iron-refractory anaemia and growth retardation (*6*). To date, 18 patients from 15 families are known to be affected by congenital atransferrinemia, making it an exceptional occurrence (*6*). Conversely, acquired (moderate) hypotransferrinemia is a relatively frequent condition; common causes include urinary loss, digestive loss, hepatic failure, and inflammation (*1*). However, very few descriptions of acquired atransferrinemia have been reported to date. These were associated with cirrhosis, haemochromatosis, nephrotic syndrome, and erythroleukemia (*8*).

**Figure 2:**
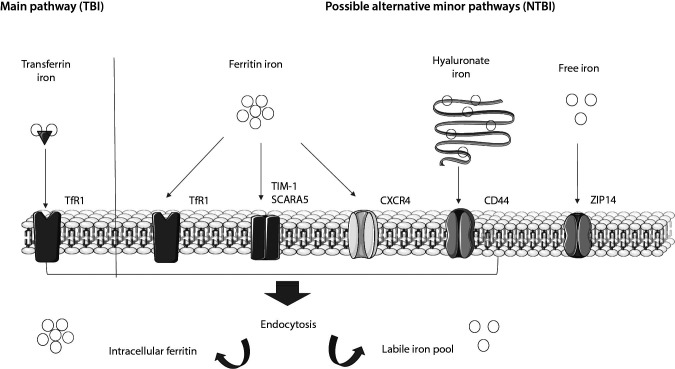
Iron uptake pathways in erythroid precursors. Physiologically, the transferrin/transferrin receptor 1 (Tf/TfR1) pathway supplies the iron required for haemoglobin synthesis. Possible minor pathways, which could play a significant role when the Tf/TfR1 pathway is affected, could include ferritin delivery mediated by TIM-1, SCARA5, TfR1 or CXCR4, or non-transferrin-bound iron (NTBI) delivery by ZIP14 or CD44. TfR1 - transferrin receptor 1. TBI - transferrin-bound iron. NTBI - non-transferrin-bound iron. Original figure designed by Raynor *et al*.

Beyond laboratory results on admission, it is interesting to discuss the relative stability of the patient’s haemoglobin concentration throughout hospitalization. One possibility is that this was achieved by repeated RBC transfusions and by the limited duration of the documented atransferrinemia (2 months), shorter than the average RBC lifespan in healthy individuals (120 days). However, several studies have shown that in patients suffering from diverse conditions, including iron-deficiency anaemia, as observed for this patient, RBC lifespan is significantly decreased (*9*). Transfused RBC lifespan is also substantially shorter than that of endogenous RBC (50-60 days) (*10*). Finally, we cannot exclude that the atransferrinemia was present in the patient before admission, as we lack laboratory data. A second possibility is that the trace amounts of Tf evidenced in the patient’s serum by capillary electrophoresis were sufficient to maintain a residual erythropoiesis. Some authors proposed this as an explaination for the persistent erythropoiesis in patients suffering from congenital atransferrinemia (*11*, *12*). A third, more intriguing possibility, is that other pathways than Tf/TfR1 were able to provide iron to erythroid precursors. Indeed, although poorly demonstrated, it has been previously shown in some models that erythroid precursors can meet their iron needs without Tf. Some of the current hypotheses for alternative iron uptake pathways are summarized in Figure 2. *In vitro,* human erythroid precursors can proliferate in Tf-free medium supplemented with ferritin (*13*). Moreover, monocyte-derived macrophages can export ferritin to human erythroid precursors in a co-culture setting, probably through exocytosis followed by receptor-mediated endocytosis (*14*). This uptake could involve TfR1, which can internalize H-ferritin in human erythroid cells *in vitro* (*15*). However, older studies have shown that this process inhibits normal hematopoiesis *in vitro* and *in vivo* (*16*, *17*). Other receptors, including scavenger receptor class A member 5 (SCARA5), T-cell immunoglobulin and mucin domain 1 (TIM-1), and C-X-C chemokine receptor type 4 (CXCR4) have been shown to internalize ferritin *in vitro*, although their expression in erythroid precursors remains to be investigated (*18*, *19*). Other possible pathways involve iron uptake from non-transferrin-bound iron (NTBI). For instance, citrate-Fe^3+^ complexes were detected in the sera of patients with haemochromatosis (*20*). T lymphocytes and reticulocytes can take up citrate-Fe^3+^ complexes *in vitro* (*21*-*23*). Erythroid precursors can also incorporate NTBI *via* plasma membrane transporters such as ZRT-, IRT-like protein 14 (Zip14) (*24*). Cluster of differentiation 44 (CD44) is expressed on erythroid precursors and internalizes iron-bound hyaluronates (*25*). A description of congenital dyserythropoietic anaemia associated with CD44 deficiency but without altered iron metabolism has been published (*26*). Finally, ferritinophagy mediated by nuclear receptor coactivator 4 (NCOA4) in erythroid island macrophages possibly plays a significant role in maintaining erythropoiesis in the absence of Tf. Indeed, NCOA4 deficiency is associated with microcytic anaemia in various models (*27*, *28*). In NCOA4-KO mice, splenic iron overload was observed, possibly pointing at defective iron release by macrophages (*28*). However, it is unclear how erythroid island macrophages could provide iron to erythroid precursors *in vivo*. Iron export from macrophages through ferroportin-1 (Fpn1) has been proposed (*29*). Mice with Fpn1-deficient macrophages displayed anaemia and peripheral iron overload. After iron export, erythroid island ceruloplasmin and Tf, or direct cell-to-cell transfer could be involved in erythroid precursors’ uptake (*30*). Finally, the aforementioned pathways could provide an additional explanation for persistant erythropoiesis in patients with congenital transferrinemia, which may not solely rely on trace amounts of serum Tf.

In conclusion, we report an exceptional occurrence of acquired transferrinemia in a patient with chronic enteropathy, related to chronic inflammation, undernutrition and liver failure. The mildness of the clinical and haematological manifestations raises questions on possible compensatory mechanisms for erythroblastic iron supply, which remain to be investigated.
